# Effects of Rhamnolipid and Microbial Inoculants on the Vermicomposting of Green Waste with *Eisenia fetida*

**DOI:** 10.1371/journal.pone.0170820

**Published:** 2017-01-25

**Authors:** Xiaoqiang Gong, Le Wei, Xin Yu, Suyan Li, Xiangyang Sun, Xinyu Wang

**Affiliations:** College of Forestry, Beijing Forestry University, Beijing, P.R. China; USDA Forest Service, UNITED STATES

## Abstract

The effects of adding the biosurfactant rhamnolipid, the lignolytic and cellulolytic fungus *Phanerochete chrysosporium*, and the free-living nitrogen-fixing bacterium *Azotobacter chrococcum* on vermicomposting of green waste with *Eisenia fetida* was investigated. The addition of rhamnolipid and/or either microorganism alone or in all combinations significantly increased *E*. *fetida* growth rate, the number of *E*. *fetida* juveniles and cocoons, the population densities of cellulolytic fungi and *Azotobacter* bacteria, and cellulase and urease activities in the vermicomposts. The quality of the final vermicompost (in terms of electrical conductivity, nutrient content, C/N ratio, humic acid content, lignin and cellulose contents, and phytotoxicity to germinating seeds) was enhanced by addition of rhamnolipid and/or microorganisms. The physical characteristics of vermicomposts produced with rhamnolipid and/or microorganisms were acceptable for agricultural application. The best quality vermicompost was obtained with the combined addition of *P*. *chrysosporium*, *A*. *chrococcum*, and rhamnolipid.

## Introduction

The city of Beijing has more than 47,000 ha of green space that annually produces more than 2.37 million tons of green waste [1]. The green waste has been traditionally eliminated by deposition in landfills or by incineration but these disposal methods cause problems. The deposition of green waste in landfills subtracts from the available cultivated land and also pollutes surface and ground water [2]. Incineration of green waste leads to the formation of the greenhouse gases carbon dioxide and methane [3]. In addition, both methods result in the loss of green waste as a biomass resource. Therefore, safe and environmentally friendly methods of green waste disposal are needed.

Vermicomposting is a promising technology for the treatment of solid organic waste [4]. Vermicomposting involves the bio-oxidation and stabilization of organic material under aerobic and mesophilic conditions through the combined action of earthworms and microorganisms [5]. There are few reports, however, of the use of vermicomposting for treatment of green wastes. Compared with other types of organic wastes, green waste contains higher amounts of lignin and cellulose, which might lead to poor compost quality [6, 7].

Microbial activity plays a key role in organic matter biodegradation and in nutrient transformation during vermicomposting [8]. Inoculation of suitable microorganisms could accelerate the vermicomposting process and improve compost quality. The white-rot fungus *Phanerochete chrysosporium* is one of the most efficient microorganisms at degrading lignin and cellulose [9]. El-Haddad et al. [10] observed that inoculation with *P*. *chrysosporium* accelerated lignin and cellulose degradation during vermicomposting of rice straw and also improved the quality of the vermicompost product. Inoculation with nitrogen-fixing bacteria could also improve compost quality. Das et al. [11] reported that inoculation with the nitrogen-fixing bacterium *Azotobacter chrococcum* during vermicomposting increased the nitrogen content of the final product.

Recent studies have documented that the application of a biosurfactant improves composting efficiency and the quality of the final product during the aerobic composting of municipal solid waste [12], garden waste [13], and agricultural waste [14]. Rhamnolipids are widely used, commercially available biosurfactants. Rhamnolipids, which are anionic compounds mainly produced by *Pseudomonas* spp., have high biodegradability, biocompatibility, and surfactant activity [15]. Liang et al. [16] found that addition of a rhamnolipid stimulated microbial enzymatic activity and increased microbial biomass during the composting of rice straw. Jahanshahet al. [17] reported that a rhamnolipid reduced surface tension, promoted microbial growth, and accelerated the degradation of organic matter during the composting of municipal waste. However, the effects of rhamnolipids on the vermicomposting of green waste are unknown.

The current research investigated the effects of a rhamnolipid biosurfactant and microbial inoculants (*P*. *chrysosporium* and *A*. *chrococcum*) on the vermicomposting of green waste. The earthworm *Eisenia fetida* was selected for this study because it tolerates wide ranges of pH, temperature, and moisture content [18].

## Materials and Methods

### Ethics statement

The experiment was carried out in our scientific research greenhouse which is owned by our institute, therefore, no specific permissions were required for these locations/activities. We also confirm that the studies did not involve endangered or protected species.

### Green waste, *E*. *fetida*, and rhamnolipid

The green waste used in this study was obtained from a municipal green waste treatment plant in Chaoyang District, Beijing, China, and consisted of fallen leaves, grass clippings, and branch cuttings. The physico-chemical characteristics of the raw material were as follows: pH, 6.21; electrical conductivity (EC), 0.41 mS cm^-1^; total organic carbon (TOC), 487.3 g kg^-1^; total nitrogen (TN), 13.0 g kg^-1^; total phosphorus (TP), 2.1 g kg^-1^; total potassium (TK), 2.6 g kg^-1^; carbon:nitrogen ratio (C/N), 37.4; humic acid content, 3.3%; cellulose content, 57.2%; and lignin content, 26.7%.

Adult (clitellate) *E*. *fetida* earthworms of uniform size were obtained from a commercial earthworm breeding farm in Shunyi District, Beijing, China. Rhamnolipid in the form of a brown, water-soluble paste was purchased from HuzhouZijin Biotechnology Co., Ltd. Zhejiang, China. (Zhejiang, China). Its major components were di-rhamnolipid (C_32_H_58_O_13_) and mono-rhamnolipid (C_26_H_48_O_9_) at a mass ratio of 1:2. The critical micelle concentration of rhamnolipid was determined to be 50 mg L^-1^.

### Microbial source

Pure cultures of *P*. *chrysosporium* (a lignolytic and cellulolytic fungus) and *A*. *chrococcum* (a free-living, nitrogen-fixing bacterium) were obtained from the China General Microbiological Culture Center (CGMCC), Beijing, China. The preservation numbers of *P*. *chrysosporium* and *A*. *chrococcum* in CGMCC are 5.0776 and 1.0151, respectively. Before being used to inoculate compost, *P*. *chrysosporium* was cultivated in 500 ml conical flasks containing 250 ml of modified potato dextrose broth (distilled H_2_O, 1000 ml; peeled potato, 200 g; dextrose, 20.0 g; KH_2_PO_4_, 3.0 g; MgSO_4_·7H_2_O, 1.5 g; vitamins, trace; pH adjusted to 7.0) at 28°C and 170 rpm for 7 days. *A*. *chrococcum* was grown in 500 ml conical flasks containing 250 ml of modified Jensen's nitrogen-free medium (distilled H_2_O, 1000 ml; sucrose, 20.0 g; K_2_HPO_4_, 1.0 g; MgSO_4_, 0.5 g; NaCl, 0.5 g; FeSO_4_, 0.1 g; Na_2_MoO_4_, 0.005 g; CaCO_3_, 2.0 g; pH adjusted to 7.2) at 30°C and 170 rpm for 7 days. The final concentrations of the *P*. *chrysosporium* and *A*. *chrococcum* strains were 1×10^9^ colony forming units (CFU) ml^-1^ and 1×10^8^ CFU ml^-1^, respectively.

### Experimental design

An experiment was conducted in a greenhouse at Beijing Forestry University Forest Science Company Limited Nursery, Beijing, China. The temperature in the greenhouse during the experiment ranged from 26.2 to 28.8°C. The experiment included a pre-composting phase (21 days) and a subsequent vermicomposting phase (60 days).

During the pre-composting phase, the green waste was shredded into pieces of approximately 5 mm. Thereafter, 0.25 m^3^ of the shredded material was loaded into polyethylene vermicomposting containers (0.6 m wide, 0.8 m long, and 0.65 m high). The bottom of each container had 20 holes (10 mm diameter) for drainage; these holes were covered with 1 mm plastic mesh to prevent earthworm escape during the vermicomposting phase. Urea was added to the raw material to adjust the initial C/N ratio to 25, and water was added to adjust the moisture content to 60–70%; this moisture content was maintained by adding water when necessary throughout the pre-composting phase.

After 21 days of pre-composting, 1600 adult *E*. *fetida* with an average fresh weight of 186 mg per individual were added to each container; this density corresponded to the optimal worm stocking density suggested by Chan et al. [19]. The moisture content was maintained at 65–70% by periodic sprinkling of distilled water throughout the vermicomposting phase.

*P*. *chrysosporium*, *A*. *chrococcum*, and rhamnolipid were added to designated containers at zero day and 30^th^ day of vermicomposting in the following eight combinations: CK (control, nothing added); P (*P*. *chrysosporium* alone); A (*A*. *chrococcum* alone); PA (*P*. *chrysosporium* + *A*. *chrococcum*); R (rhamnolipid alone); RP (rhamnolipid + *P*.*chrysosporium*); RA (rhamnolipid *+ A*. *chrococcum*); and RPA (rhamnolipid*+ P*. *chrysosporium*+ *A*. *chrococcum*).

Rhamnolipid was dissolved in water (1:100 w/v) and then added to the materials at a concentration of 15 g kg^-1^ (the original fluid) of dry green waste. For each 1 kg of dry waste, 20 ml of *P*. *chrysosporium*, 20 ml of *A*. *chrococcum*, and 40 ml of *P*. *chrysosporium* and *A*. *chrococcum* combination (1:1 v/v) were inoculated, respectively.

After all the treatments were finished, materials were evenly mixed. The experiment had a completely randomized design with three replicate containers per treatment. During the pre-composting and vermicomposting phases, the material was manually turned every 7 days to provide aerobic conditions and to ensure uniform decomposition.

At the end of the experiment, the vermicomposting each container was homogeneously mixed, and 1 kg of vermicompost was taken from each container to determine earthworm growth, reproduction, and cocoon production. To accomplish this, earthworms, cocoons, and hatchlings were separated from the vermicomposts by hand and were counted and weighed after they were washes with distilled water.

After adults, cocoons, and hatchlings had been removed, nine samples were collected randomly from each container and were then mixed to give a composite sample of about 400 g per container. Each composite sample was divided into two parts. One part was kept fresh for assessment of cellulolytic fungal and *Azotobacter* bacteria population densities, and cellulase and protease activities; fresh samples were also used for a seed germination test. The other part was dried at 65°C and then used to determine physical characteristics, pH, and EC. After it was finely pulverized, dried sample was also used to determine of contents of TOC, TKN, TP, TK, lignin, cellulose, and humic acid.

### Physical-chemical analysis

Bulk density, total porosity and aeration porosity of the final vermicomposts were determined by the ring knife method described by Tian et al. [20]. Particle size of the final vermicomposts was estimated as per the procedure described by Fornes et al. [21]. The pH and EC of the samples were measured in a 1:10 (w/v) aqueous suspension (distilled water) using a pH meter (Starter 3C; Ohaus Instrument (Shanghai) Co., Ltd., Shanghai, China) and a conductivity meter (DDS-11A; Shanghai Leici-Chuangyi Instrument Co., Ltd., Shanghai, China).

The method described by Juradol et al. [22] was used for the detection of cellulase activity, based on the colourimetric estimation of the glucose released in the reaction with 3, 5-dinitrosalicylic acid (DNS) at 37°C for 2 h. Urease activity was measured following the method of Juradol et al. [22]. The population densities of culturable cellulolytic fungi and *Azotobacter* bacteria were determined using the standard dilution spread-plate method described by Pramanik et al. [23] and Kumar and Singh [24]. One gram of fresh sample was stirred with 100 ml sterile distilled water in a conical flask and the supernatant was serially diluted 10^3^, 10^4^, 10^5^, and 10^6^ times to estimate the population of cellulolytic fungal and *Azotobacter* bacteria in asparagine medium (distilled H_2_O, 1000 ml; glucose, 10 g; asparagines, 1 g; KH_2_PO_4_, 3 g; MgSO_4_·7H_2_O, 0.5 g; vitamin B1, trace; pH adjusted to 7.0) and Jensen's medium (distilled H_2_O, 1000 ml; sucrose, 20.0 g; K_2_HPO_4_, 1.0 g; MgSO_4_, 0.5 g; NaCl, 0.5 g; FeSO_4_, 0.1 g; Na_2_MoO_4_, 0.005 g; CaCO_3_, 2.0 g; pH adjusted to 7.2), respectively. Plates were incubated for 24 h (bacteria) and 72 h (fungi) to count the CFUs of microbes.

TOC was measured using the wet oxidation method proposed by Yeomansand Bremner [25]. TKN was determined by the Kjeldahl method as described by Barrington et al. [26] using an automatic Kjeldahl analyzer (KDY-9830; Beijing Tongrunyuan Mechatronics Technology Co., Ltd., Beijing, China). The TP and TK contents were determined after digesting a 0.1 g sample with 98% (v/v) sulfuric acid and 30% (v/v) hydrogen peroxide. TP was analyzed by the anti-Mo-Sb spectrophotometry method according to Li et al. [27] using a UV spectrophotometer (UV-120-02; Shimadzu Scientific Instruments, Kyoto, Japan). TK was analyzed by flame photometry using a flame photometer (425; Spring Instrument Equipment Co., Ltd., Shanghai, China). Lignin was measured using the 72% (v/v) H_2_SO_4_ method outlined by Liu [28]. Cellulose was measured by the HNO_3_-ethanol method described by Liu [28]. Humic acid content was estimated following the methods suggested by Pramanik et al. [29]. The C/N values were calculated using the measured values of TOC and TKN.

### Phytotoxicity test

The seed germination index (GI) was used to assess the phytotoxicity of the final compost products. A 10-g quantity of the compost from each replicate container was placed in 100 ml of distilled water. The mixture was shaken at 160 rpm on a reciprocal shaker for 30 min at room temperature and was then passed through a piece of qualitative filter paper to obtain an aqueous extract. Twenty seeds of pakchoi (*Brassica rapa* L., Chinensis group) were placed evenly in a filter paper-lined Petri dish (9 cm diameter). The seeds in the dish were then moistened with 10 ml of the aqueous extract or distilled water (control). The Petri dishes were kept at 25°C in a constant temperature incubator without light for 3 days. The germination index (GI) was then calculated according to the following equation:

$$\text{GI}\left(\text{\%}\right)=\frac{G1\times L1}{G2\times L2}\times 100\%$$

where G1 is the number of seeds germinated in the compost extract, L1 is the average root length in the compost extract, G2 is the number of seeds germinated in distilled water, and L2 is the average root length in distilled water.

### Statistical analysis

Two-way ANOVAs were used to assess the effects of rhamnolipid, the microbial inoculants (treated as one main factor), and their interaction on earthworm growth and fecundity and on the physical, chemical, and biological characteristics of the vermicomposts. When an ANOVA was significant, LSD post-hoc tests were used to compare the eight means at *P*<0.05 [23, 30]. All statistical analyses were performed using the SPSS 18.0.

## Results and Discussion

### Effects of microbial inoculants and rhamnolipid on the growth and reproduction of *E*. *fetida*

The microbial inoculants, rhamnolipid, and their interaction significantly affected the earthworm growth rate and the production of juveniles (Table 1). The earthworm growth rate and juvenile production were increased by all additive treatments (by all treatments involving addition of rhamnolipid or one or both microorganisms) and were highest with RPA and lowest with CK.

**Table 1:** Effects of the microbial inoculants and rhamnolipid on the growth and reproduction of the earthworm *Eisenia fetida*.

Treatment^a^	Earthworm growth rate (mg worm^-1^ day^-1^)	Cocoon production (number kg^-1^)	Juvenile production (number kg^-1^)
CK	0.96±0.09 e	20±2 e	60±5 g
P	1.59±0.06 d	33±3 cd	89±2 ef
A	1.43±0.02 d	27±1 de	80±3 f
PA	1.87±0.05 c	35±1 c	99±2 e
R	1.88±0.06 c	39±4 c	114±4 d
RP	2.18±0.01 b	58±4 a	156±5 b
RA	1.93±0.06 c	50±2 b	136±3 c
RPA	2.48±0.11 a	66±3 a	180±7 a
Microbial inoculants(MI)^b^	46.4***	25.3***	54.8***
Rhamnolipid (R)	195.2***	180.1***	465.7***
MI×R	3.7*	1.6ns	4.5*

^a^CK (control; nothing added); P (*P*. *chrysosporium* alone); A (*A*. *chrococcum* alone); PA (*P*. *chrysosporium* + *A*. *chrococcum*); R (rhamnolipid alone); RP (rhamnolipid + *P*. *chrysosporium*); RA (rhamnolipid *+ A*. *chrococcum*); and RPA (rhamnolipid*+ P*. *chrysosporium*+ *A*. *chrococcum*). Values are means (± SD, n = 3). Means in the same column followed by different letters are significantly different at *P* < 0.05 according to the LSD test.

^b^The effects (*F* values) of the microbial inoculants, rhamnolipid, and their interaction are indicated in the bottom three rows. ns, *, *** indicate not significant and statistically significant at *P* < 0.05 and < 0.001, respectively.

The microbial inoculants and rhamnolipid addition also significantly affected cocoon production (Table 1). Cocoon production was increased by all additive treatments except A and was highest with RP and RAP and lowest with CK and A.

The results indicated that addition of the biosurfactant rhamnolipid or the microorganisms individually or in combination significantly enhanced earthworm growth rate and earthworm production of juveniles and cocoons. Bonkowskiet al. [31] indicated that microorganisms are considered to be an important food source for earthworms and that earthworms can selectively digest them during vermicomposting. The increased growth of *E*. *fetida*in response to addition of microbial inoculants may be explained by the fact that the inoculants provided additional food resources. Similar results were obtained by Rahul and Shweta [32]. Moreover, Slizovskiy et al. [33] suggested that rhamnolipid can reduce the bioaccumulation of toxic chemicals in *Eisenia fetida*, which in turn could result in increased earthworm growth.

### Effects of the microbial inoculants and rhamnolipid on microbial population densities and enzymatic activities

Microbial inoculants and rhamnolipid significantly affected the population densities of cellulolytic fungi and *Azotobacter* bacteria (Table 2); the interaction was significant for *Azotobacter* bacteria but not for cellulolytic fungi. Population densities of cellulolytic fungi were increased by all additive treatments and were highest with RP and RPA and lowest with CK. Population densities of *Azotobacter* bacteria were also increased by all additive treatments and were highest with RPA and lowest with CK.

**Table 2:** Effects of the microbial inoculants and rhamnolipid on the population densities of cellulolytic fungi and *Azotobacter* bacteria and on cellulase and urease activities in vermicomposts.

Treatment^a^	Numbers of cellulolytic fungi (10^6^)	Numbers of *Azotobacter* bacteria (10^4^)	Cellulase activity (μg glucose g^-1^ h^-1^)	Urease activity (μg NH_3_ g^-1^ h^-1^)
IM^c^	5.8±0.5 g	5.1±0.3 h	850±42 g	395±16 h
CM^d^	10.6±0.6 f	8.1±0.4 g	1226±22 f	1147±41 g
CK	19±2 e	14±1 f	1828±37 e	1621±18 f
P	77±3 cd	51±4 e	2154±17 c	1889±21 e
A	51±1 d	68±2 d	1988±31 d	2130±42 d
PA	86±6 c	66±2 d	2141±32 cd	2289±26 d
R	177±9 b	115±8 c	2832±46 b	2810±72 c
RP	249±19 a	120±5 c	3240±108 a	3041±56 c
RA	176±11 b	164±3 b	2812±55 b	3976±94 b
RPA	250±10 a	178±5 a	3272±34 a	4373±175 a
Microbial inoculants (MI)^b^	27.8***	71.6***	29.4***	83.2***
Rhamnolipid(R)	539.6***	884.7***	778.0***	771.0***
MI×R	2.4ns	8.4**	3.5*	17.3***

^a^Treatment abbreviations are explained in Table 1. Values are means (± SD, n = 3). Means in the same column followed by different letters are significantly different at *P* < 0.05 according to the LSD test.

^b^The effects (*F* values) of the microbial inoculants, rhamnolipid, and their interaction are indicated in the bottom three rows. ns, *, **, *** indicate not significant and statistically significant at *P* < 0.05, < 0.01, and < 0.001, respectively.

^c^Initial material.

^d^Pre-composted material (21st day after composting).

The increase in numbers of cellulolytic fungi and *Azotobacter* bacteria could be explained by their addition to the substrate. In addition, rhamnolipid can cause the dispersion of the organic material to the aqueous phase, which can enhance the mass transfer of the organic material to the microorganisms or increase the concentration of the organic molecules that can be directly assimilated by the microorganisms [34].

Cellulase and urease activities were significantly affected by the microbial inoculants, rhamnolipid, and their interaction (Table 2). Cellulase activity was increased by all additive treatments and was highest with RP and RPA and lowest with CK. Cellulase activity was positively correlated with the population density of cellulolytic fungi (*r* = 0.969) and with the humic acid content of the vermicomposts (*r* = 0.906) and was negatively correlated with cellulose and lignin contents of the vermicomposts (*r* = -0.917 and *r* = -0.932, respectively). Urease activity was also increased by all additive treatments and was highest with RPA and lowest with CK. Urease activity was positively correlated with the population density of *Azotobacter* bacteria (*r* = 0.972) and with the nitrogen content (*r* = 0.779) and the humic acid content (*r* = 0.887) of the vermicomposts. The positive correlation between cellulase activity, urease activity, and humic acid content suggests that humic acids might be responsible for preserving these enzymes as humic-enzyme complexes in the vermicomposts [35, 36].

In this study, the high and positive correlations between enzyme activities and microbial numbers suggest that the enhanced enzyme activity in the vermicomposts was due to enhanced numbers of microbes. The positive effect of rhamnolipid on total enzyme activities can be explained by an increase in the permeability of cell membranes and thereby an increase in the rate at which enzymes are excreted from microbial cells [37]. Furthermore, Kim et al. [38] and Wang et al. [39] indicated that rhamnolipid can reduce enzyme degradation and inactivation by reducing enzyme contact with the air–liquid interface.

### Effects of the microbial inoculants and rhamnolipid on the physical properties of vermicompost

Microbial inoculants, rhamnolipid, and their interaction significantly affected bulk density (Table 3). Bulk density was increased by all additive treatments and was lowest with CK and highest with RPA, RP, and RA. The higher bulk densities with the latter treatments could be due to their enhancement of the degradation rate. Similar responses to rhamnolipid addition were reported by Zhang et al. [7] and to *P*. *chrysosporium* addition by Pratibha et al. [40]. Abad et al. [41] suggested that compost used as a growing substrate should have a bulk density < 0.40 g cm^−3^. In this study, the bulk densities of all vermicomposts were < 0.40 g cm^−3^.

**Table 3:** Effects of the microbial inoculants and rhamnolipid on the physical properties of vermicompost.

Treatment^a^	Bulk density (g cm^-3^)	Particle size (dg ^c^) (mm)	Total porosity (%)	Aeration porosity (%)
CK	0.277±0.002 f	2.12±0.006 a	75.87±0.49 a	28.81±0.63 a
P	0.303±0.003 d	2.00±0.003 c	71.72±0.27 c	22.12±0.74 c
A	0.291±0.003 e	2.03±0.005 b	73.64±0.46 b	24.67±0.34 b
PA	0.313±0.000 c	1.99±0.003 cd	71.28±0.17 c	20.50±0.39 d
R	0.317±0.006 bc	1.97±0.002 d	70.79±0.14 c	17.90±0.21 e
RP	0.329±0.002 a	1.89±0.010 f	69.17±0.67 d	14.98±0.60 fg
RA	0.324±0.002 ab	1.91±0.011 e	69.62±0.33 d	16.46±0.67 ef
RPA	0.330±0.004 a	1.87±0.008 f	69.19±0.27 d	14.89±0.24 g
Microbial inoculants (MI)^b^	23.5***	113.5***	27.1***	47.7***
Rhamnolipid (R)	169.7***	668.4***	158.3***	478.7***
MI×R	4.9 *	3.7*	6.3**	9.4**

^a^Treatment abbreviations are explained in Table 1. Values are means (± SD, n = 3). Means in the same column followed by different letters are significantly different at *P* < 0.05 according to the LSD test.

^b^The effects (*F* values) of the microbial inoculants, rhamnolipid, and their interaction are indicated in the bottom three rows. ns, *, **, *** indicate not significant and statistically significant at *P* < 0.05, < 0.01, and < 0.001, respectively.

^c^dg: geometric mean diameter.

Particle size, expressed as the geometric mean diameter (dg), significantly affected by the addition of rhamnolipid, the addition of microorganisms, and by their interaction (Table 3). The particle size was decreased by all additive treatments and was lowest with RPA and RP and highest with CK.

The microbial inoculants, rhamnolipid, and their interaction significantly affected total porosity and aeration porosity (Table 3). Total porosity and aeration porosity were reduced by all additive treatments and were lowest with RPA, RP, and RA and highest with CK. Total porosity values between 54 and 96% and water-holding porosity values between 36 and 77% are generally considered acceptable for crop cultivation [42]. Total porosity and aeration porosity for all vermicomposts in the current study were therefore suitable for crop cultivation.

### Effects of the microbial inoculants and rhamnolipid on the chemical properties of vermicompost

pH was significantly affected by the addition of rhamnolipid but not by the addition of microorganisms or by the interaction between the two factors (Table 4). The pH value was lower with RP and RPA than with CK. Rhamnolipid application could have decreased the pH by increasing the microbial population and thereby accelerating organic matter decomposition and the release of organic acids [14]. At the end of the experiment, the pH values of all vermicomposts were within the satisfactory range (7.0 to 8.5) for agricultural use [43].

**Table 4:** Effects of the microbial inoculants and rhamnolipid on the chemical properties of vermicompost.

Treatment^a^	pH	EC (mS cm^-1^)	TOC (g kg^-1^)	TN (g kg^-1^)	TP (g kg^-1^)	TK (g kg^-1^)
CK	8.28±0.02 a	0.870±0.003 f	367.4±4.9 a	17.00±0.14 e	4.37±0.06 e	3.30±0.06 c
P	8.24±0.03 ab	0.982±0.004 de	345.4±3.4 bc	17.76±0.03 d	4.86±0.03 cd	3.78±0.05 b
A	8.23±0.01 ab	0.946±0.003 e	351.5±2.1b	18.80±0.17 b	4.55±0.04 d	3.33±0.02 c
PA	8.23±0.03 ab	0.995±0.013 de	340.0±0.6 c	18.69±0.18 bc	4.87±0.05 b	3.81±0.05 b
R	8.22±0.04 ab	1.032±0.017 cd	323.6±2.0 d	17.67±0.04 d	4.74±0.03 c	3.67±0.05 b
RP	8.20±0.03 b	1.205±0.042 b	306.9±3.5 ef	18.16±0.06 cd	5.18±0.07 a	4.46±0.12 a
RA	8.21±0.03 ab	1.098±0.022 c	316.2±2.6 de	19.86±0.36 a	4.86±0.05 bc	3.85±0.07 b
RPA	8.19±0.01 b	1.323±0.048 a	300.7±6.9 f	20.11±0.36 a	5.20±0.06 a	4.39±0.08 a
Microbial inoculants (MI)^b^	1.25ns	25.5***	17.0***	48.60***	42.97***	47.50***
Rhamnolipid (R)	5.02 *	147.3***	223.0***	36.35***	86.05***	123.64***
MI×R	0.18ns	5.2*	0.4ns	2.29ns	0.15ns	1.72ns
Treatment^a^	C/N ratio	Humic acid (%)	Ligin (%)	Cellulose (%)	GI	
CK	21.62±0.43 a	7.02±0.23 d	21.99±0.38 a	32.47±0.54 a	86.3±1.1 f	
P	19.45±0.17 b	8.08±0.15 c	18.53±0.30 c	28.26±0.34 c	97.0±1.0 de	
A	18.70±0.25bc	8.06±0.08 c	20.35±0.15 b	31.14±0.87 b	95.6±1.0 e	
PA	18.20±0.20 c	8.21±0.08 c	17.45±0.08 d	27.34±0.13 c	100.9±2.6 d	
R	18.31±0.08 c	8.83±0.08 b	16.37±0.16 e	25.70±0.26 d	109.3±2.0 c	
RP	16.90±0.14 d	9.20±0.10 a	14.53±0.15 f	23.96±0.17 e	119.4±1.3 b	
RA	15.93±0.16 e	9.24±0.07 a	15.94±0.14 e	24.52±0.21 de	114.1±1.1 c	
RPA	14.97±0.60 f	9.54±0.07 a	14.10±0.11 f	22.33±0.07 f	130.5±1.9 a	
Microbial inoculants (MI)^b^	46.3***	23.99***	112.7***	42.03***	42.7***	
Rhamnolipid (R)	191.9***	261.85***	895.2***	386.28***	421.2***	
MI×R	0.7ns	3.48*	10.8***	4.43*	4.1*	

^a^Treatment abbreviations are explained in Table 1. Values are means (± SD, n = 3). Means in the same column followed by different letters are significantly different at *P* < 0.05 according to the LSD test.

^b^The effects (*F* values) of the microbial inoculants, rhamnolipid, and their interaction are indicated in the bottom three rows. ns, *, *** indicate not significant and statistically significant at *P* < 0.05 and < 0.001, respectively.

EC values were significantly affected by the addition of rhamnolipid, the addition of microorganisms, and by their interaction (Table 4). The EC value was increased by all additive treatments and was highest with RPA and lowest with CK. The EC value of all treatments was < 3dS m^-1^, a level which is considered a safety threshold for composts that are applied to soil [44].

The TOC content of the vermicomposts was significantly affected by addition of rhamnolipid and microorganisms but not by their interaction (Table 4). The TOC content was reduced by all additive treatments and was lowest with RAP and RP and highest with CK. The decrease in TOC content in response to inoculation with *P*. *chrysosporium* was probably due to the utilization and breakdown of complex organic matter by the fungus [45]. The decrease in TOC content in response to addition of rhamnolipid could be due to a surfactant-induced increase in microbial growth [46], which would have accelerated the degradation of organic matter.

TN content was significantly affected by rhamnolipid addition and microbial inoculation but not by their interaction (Table 4). TN content was increased by all additive treatments and was highest with RPA and RA and lowest with CK. Kumar and Singh [24] reported that the production of nitrogenase by *A*. *chrococcum* might have contributed to an increase in TN content during vermicomposting. Rhamnolipid may have increased the nitrogen content by enhancing the activity of nitrogen-fixing bacteria, and additionally accelerated the decomposition of organic carbon and consequently increased the total nitrogen content.

TP and TK were significantly affected by rhamnolipid addition and microbial inoculation but not by their interaction (Table 4). TP and TK were increased by most additive treatments and were highest with RPA and RP. TP was lowest with CK, and TK was lowest with CK and A.

The C/N ratio was significantly affected by rhamnolipid addition and microbial inoculation but not by their interaction (Table 4). The C/N ratio was significantly decreased by all additive treatments. Van Heerden et al. [47] suggested that a C/N ratio < 20 indicates that the compost is mature, and that a ratio < 15 is preferred for composts used in agronomy. In the current study, the final C/N ratios were < 20 in all additive treatments, but the ratio was < 15 only with RPA. Based on C/N ratios, the combined addition of rhamnolipid *+ P*. *chrysosporium* + *A*. *chrococcum* (RPA) resulted in the most suitable compost for agronomic use.

Humic acid content was significantly affected by rhamnolipid addition, microbial inoculation, and their interaction (Table 4). Humic acid content was increased by all additive treatments and was highest with RPA and was lowest with CK. The humic acid content may have been greater in the additive treatments because microbial inoculation and rhamnolipid addition may have accelerated the conversion of organic matter into humic-like substances.

Lignin and cellulose content were significantly affected by rhamnolipid addition, microbial inoculation, and their interaction (Table 4). Lignin and cellulose contents were reduced by all additive treatments. Lignin content was lowest with RPA and RP and was highest with CK. Cellulose content was lowest with RPA and highest with CK. Shi et al. [48] reported that surfactants have both hydrophobic and hydrophilic heads and can therefore affect the surface properties of cellulose and make it more accessible to enzymatic hydrolysis. Singh and Sharma [49] suggested that the production of cellulose- and lignin-degrading enzymes by added microbes can accelerate the degradation of lignocellulose.

GI values were significantly affected by rhamnolipid addition, microbial inoculation, and their interaction (Table 4). The GI value was increased by all additive treatments. Zucconi et al. [50] suggested that GI values > 80% indicate that composts are mature and not phytotoxic. All of the final vermicomposts in the current study had GI values > 80%, suggesting that they were mature and not phytotoxic. The GI value was highest with RPA and lowest with CK.

## Conclusion

The results of the present study indicate that the efficiency of vermicomposting and the quality of the vermicompost were highest with the combined addition of rhamnolipid, *P*. *chrysosporium*, and *A*. *chrococcum*. This optimal combination enhanced *E*. *fetida* growth and fecundity during vermicomposting, increased microbial numbers and enzymatic activities, accelerated the decomposition of lignin and cellulose, increased the nutrient concentrations in the vermicomposts, and increased the GI value. The combination also resulted in a vermicompost with physical characteristics that were in the optimal ranges for agricultural use. Based on these results, we suggest that vermicomposting of green waste can be enhanced by the combined addition of rhamnolipid, *P*. *chrysosporium*, and *A*. *chrococcum*.
